# IL-27 Regulates IL-18 Binding Protein in Skin Resident Cells

**DOI:** 10.1371/journal.pone.0038751

**Published:** 2012-06-27

**Authors:** Miriam Wittmann, Rosella Doble, Malte Bachmann, Josef Pfeilschifter, Thomas Werfel, Heiko Mühl

**Affiliations:** 1 Leeds Institute of Molecular Medicine, LMBRU LTHT, Division of Rheumatic and Musculoskeletal Disease, University of Leeds, Leeds, United Kingdom; 2 Centre for Skin Sciences, School of Life Sciences, University of Bradford, Bradford, United Kingdom; 3 Institute of Molecular and Cellular Biology, Faculty of Biological Sciences, University of Leeds, Leeds, United Kingdom; 4 Pharmazentrum Frankfurt/ZAFES, University Hospital Goethe-University Frankfurt, Frankfurt am Main, Germany; 5 Division of Immunodermatology and Allergy Research, Department of Dermatology, Hannover Medical School, Hannover, Germany; Institut Jacques Monod, France

## Abstract

IL-18 is an important mediator involved in chronic inflammatory conditions such as cutaneous lupus erythematosus, psoriasis and chronic eczema. An imbalance between IL-18 and its endogenous antagonist IL-18 binding protein (BP) may account for increased IL-18 activity. IL-27 is a cytokine with dual function displaying pro- and anti-inflammatory properties. Here we provide evidence for a yet not described anti-inflammatory mode of action on skin resident cells. Human keratinocytes and surprisingly also fibroblasts (which do not produce any IL-18) show a robust, dose-dependent and highly inducible mRNA expression and secretion of IL-18BP upon IL-27 stimulation. Other IL-12 family members failed to induce IL-18BP. The production of IL-18BP peaked between 48–72 h after stimulation and was sustained for up to 96 h. Investigation of the signalling pathway showed that IL-27 activates STAT1 in human keratinocytes and that a proximal GAS site at the IL-18BP promoter is of importance for the functional activity of IL-27. The data are in support of a significant anti-inflammatory effect of IL-27 on skin resident cells. An important novel property of IL-27 in skin pathobiology may be to counter-regulate IL-18 activities by acting on keratinocytes and importantly also on dermal fibroblasts.

## Introduction

IL-27 is a member of the IL-12 family of cytokines and has been described to have opposing actions in inflammation. Both IL-27 receptor subunits WSX-1 and gp130 participate in signalling upon binding of IL-27 [1], [2]. IL-27 consists of two subunits, EBV-induced gene 3 (EBI3) and p28 [3], and has been shown to possess unique features in the IL-12 family such as upregulation of the high affinity IL-12R expression important for Th1 lineage polarisation [2], [3], [4], [5] or priming of antigen presenting cells (APC) for IL-23 production [6]. In human macrophages, monocytes and keratinocytes IL-27 has been shown to have a pro-inflammatory effect through the induction of CXCL10 [6], [7]. This property of IL-27 has been proposed to be of significance for the inflammatory course of eczema [7] and psoriasis [8]. CXCL10 produced by skin resident cells attracts CXCR3 expressing, predominantly IFNγ producing cells.

However, in murine models IL-27 has been shown to have anti-inflammatory effects in later stages of infection [9], [10], [11], [12]. IL-27−/− mice have been shown to be more susceptible to experimental autoimmune encephalomyelitis [11] and MRL/lpr mice overexpressing WSX-1 show reduced lupus like symptoms [12]. IL-27 has been described to inhibit Th17 differentiation in a signal transducer and activator of transcription 1 (STAT1)-dependent but IFNγ-independent manner in murine models [11]. Furthermore, IL-27 seems to stimulate murine (but not human [6], [13]) cells for IL-10 production.

IL-18 is a member of the IL-1 family and is known to have potent pro-inflammatory effects by initiating an inflammatory cytokine cascade [14], [15], [16], [17]. It supports differentiation and activation of either Th1 or Th2 cells depending on the surrounding cytokine environment and is recognised as an important regulator of both innate and acquired immunity [15], [18]. Mice deficient in IL-18 show a largely reduced IFNγ production, NK cell activity [19] and reduced chronic inflammation and airway remodelling in asthma models [20].

A number of publication have described that high levels of IL-18 and/or IL-18R [21] are expressed in lesional skin of chronic inflammatory diseases such as psoriasis and cutaneous lupus erythematosus (CLE) [22], [23], [24], [25]. We have previously shown that skin epithelial cells from CLE patients are more susceptible to IL-18 stimulation resulting in an increased TNFα production and TNFα dependent apoptosis. IL-18 is produced by skin resident dendritic cells as well as by the most abundant cell type of upper skin layers, the keratinocytes [26], [27], [28], [29], [30] but not by fibroblasts. An important proinflammatory property of IL-18 in the skin compartment may also be assumed due to the fact that skin-tropic viruses (e.g. Molluscum contagiosum, HPV) either produce a viral antagonist (vIL-18BP) or induce the production of endogenous IL-18BP [31], [32], [33], [34], [35]. It has been suggested that IL-18 plays an important role in chronification of inflammatory diseases [17], [20] and it contributes to the inflammation-fibrosis cascade in lung, kidney and cardiac pathologies [36], [37], [38], [39]. Stimulation of normal keratinocytes with IL-18 results in the production of CXCR3 ligands such as CXCL10 [21], [40] and increased surface expression of major histocompatibility complex (MHC) I and II [21], [23].

IL-18 binding protein (BP) [41] is an endogenous antagonist with high neutralising capacity that inhibits the action of IL-18 by preventing interaction with its cell surface receptors [14]. At a molar excess of two, IL-18BP neutralises IL-18 to >95% [42]. IFNγ has been described as an inducer for IL-18BP production in various cell types [43]. IL-18 and IL-18BP are both up-regulated in inflammatory conditions which suggest that IL-18BP acts as a negative feedback response in pathologies with high IFNγ levels. It has however been illustrated that the neutralising capacity of IL-18BP may not be sufficient and/or that the balanced expression of IL-18/IL-18BP may be dysregulated in a number of viral, inflammatory or fibrosing disorders [17], [33], [36], [37], [44].

IFNγ is so far the only described robust inducer of IL-18BP expression thereby acting in particular on diverse non-leukocytic cell types, among others colon carcinoma cells, HaCaT keratinocyte [43], [45], fibroblast-like synovial cells [46], and HepG2 cells [47]. By using DLD1 colon carcinoma cells, we have previously shown that STAT1 binding to a gamma-activated sequence (GAS) element in the IL-18BP promoter plays a pivotal role in the regulation of IL-18BP [48]. The significance of CCAATenhancer binding protein beta (CEBPbeta) that directs IL-18BP activation in hepatoma cells [47] and murine cardiomyocytes [49] remains to be fully elucidated in the context of skin resident cells.

Here we present data for a yet not described action of IL-27 on the induction and release of IL-18BP from human skin resident cells. These results may be helpful in understanding the potentially disturbed IL-18/IL-18BP balance in some inflammatory conditions as well as in the development of strategies for therapeutic induction of endogenous IL-18BP in inflammatory skin diseases such as CLE or psoriasis.

## Results

Skin resident cells are known producers of a wide range of molecules involved in microbial defence and inflammatory responses. Here we were interested in molecules which could lead to an increased expression of the potent IL-18 neutralising molecule IL-18BP. So far, IFNγ is the only described cytokine to upregulate IL-18BP in tissue resident cells including fibroblasts and epithelial cells [43], [45], [46], [47]. We analysed a number of ligands for pattern recognition receptors (Malp-2, Poly I:C, Murabutide) as well as cytokines belonging to the IL-12 family, oncostatin M, IL-1β, TNFα, curcurmin, prolactin, hydrocortisone, IL-15 and salbutamol. Some of these mediators have been chosen due to their capacity to activate c/EBP, binding sites for which have been identified in the IL-18BP promoter [47], [49].

**Figure 1:**
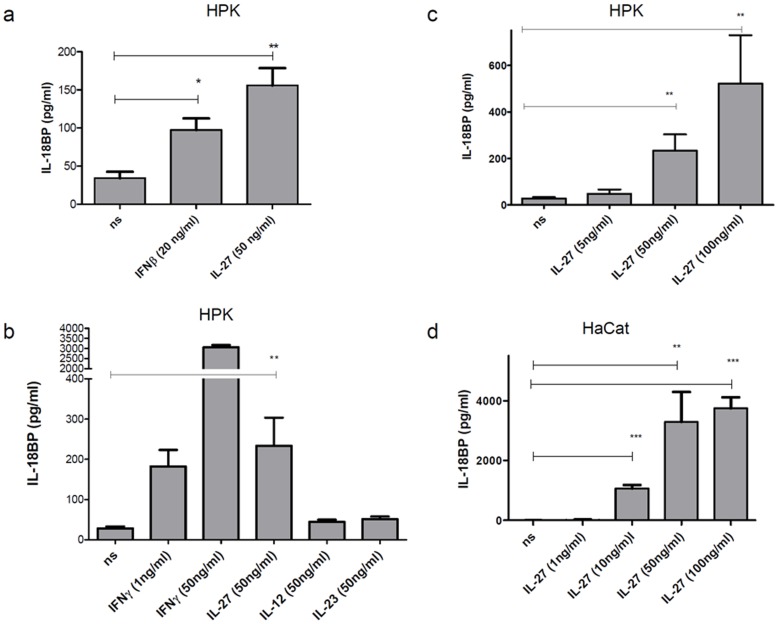
IL-27 dose-depenently induces IL-18BP secretion in human keratinocytes. Human primary keratinocytes (a, b, c) or HaCat (d) were stimulated for 48 h. Cell free supernatant was harvested and IL-18BP content was determined by Elisa. Independent experiments were performed. (a) n = 4 different experiments and donors; (b) n = 7 different experiments and donors; (c) n = 3 different experiments and donors; (d) n = 3. Mean and SEM are depicted. ns = non stimulated, HPK = human primary keratinocytes.

Apart from IFNγ the only other stimuli which markedly upregulated IL-18BP in human primary keratinocytes (HPK) were found to be IFNß and IL-27 (Figure 1A). IL-27 was the only IL-12 family member to regulate IL-18BP (Figure 1B). Since upregulation of IL-18BP by type I IFN has been observed previously [50] we chose to focus herein on IL-18BP regulation by IL-27. At a concentration of 50 ng/ml IFNγ showed approximately 100 fold stronger response than IL-27. 1 ng/ml IFNγ had an equal potency to induce IL-18BP as 50 ng/ml of IL-27 in HPK. Increasing doses of IL-27 yielded in high production of IL-18BP (Figure 1C). Secretion of protein showed no further increase at concentration higher than 100 ng/ml (data not shown). Among all cell types analysed, HPK were the only ones to produce basal levels of IL-18BP. The human keratinocyte cell line HaCat (Figure 1D) displays a very similar IL-18BP response pattern upon IL-27 (and IFNγ) stimulation as primary cells. The measured levels of IL-18BP secreted from HaCat were much higher than those seen in stimulated HPK. In HaCat cells, a dose-dependent increase of IL-18BP production could be observed for up to 200 ng/ml (mean for 200 ng/ml = 4268 pg/m, SD 1498) of IL-27 but higher concentration (e.g. 300 ng/ml) showed no further increase in the production.

**Figure 2:**
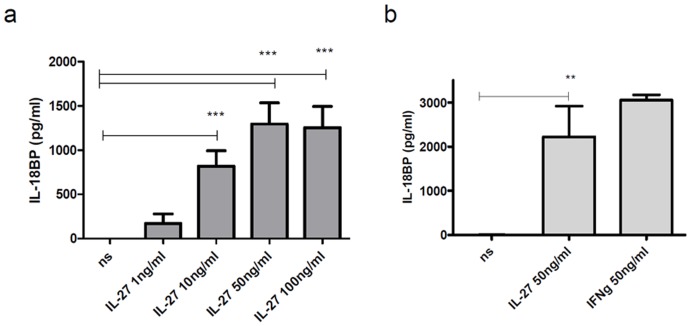
Human fibroblasts are very responsive to IL-27 stimulation. Stimulation of primary human skin fibroblasts was performed for 48 h and cell-free supernatants were analysed for IL-18BP by Elisa. Results are given as mean and SEM. (a) n = 7 (b) n = 4, ns = non stimulated.

Human primary dermal fibroblasts (Figure 2A) were found to produce unexpectedly high amounts of the IL-18BP. Fibroblasts seem very sensitive to IL-27 with around 70% of the donors responding to IL-27 concentration of 1 ng/ml (between 50 and up to 800 pg/ml IL-18BP). For fibroblasts the potency of 50 ng/ml IL-27 was in the same range as seen with equal concentration of IFNγ (Figure 2B) which was markedly different from keratinocytes. Concentration higher than 100 ng/ml (e.g. 200 and 300 ng/ml) did not further increase IL-18BP production.

**Figure 3:**
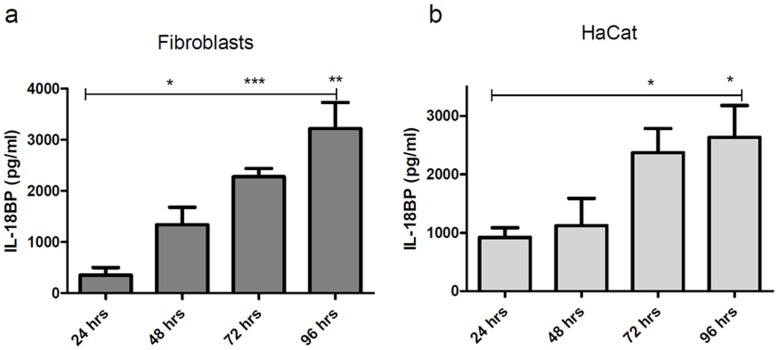
Time course of IL-18BP release by IL-27 stimulated skin cells. Fibroblasts (a) and HaCat cells (b) were cultured for up to 96 h after initial stimulation with IL-27 (50 ng/ml). Supernatants were collected at the indicated time points. Levels of IL-18BP for non stimulated cells were below the detection limit of the ELISA. n = 4 (a, b).

In fibroblasts, time kinetic experiments with IL-27 stimulated cells point to a peak in the production between 24 and 48 h of incubation and sustained production for up to 96 h (Figure 3A). The same time kinetic profile was observable for HPK (not shown) and HaCaT keratinocytes (Figure 3B). Both IL-27 and IFNγ stimulation resulted in very similar time kinetic profiles with IFNγ resulting in higher protein content in the supernatant of stimulated keratinocytes but not fibroblasts. Time periods longer than 96 h are difficult to follow up in *in-vitro* experiments. From experiments performed for 120 h we deduce that the production of IL-18BP reaches a plateau around 96 h.

**Figure 4:**
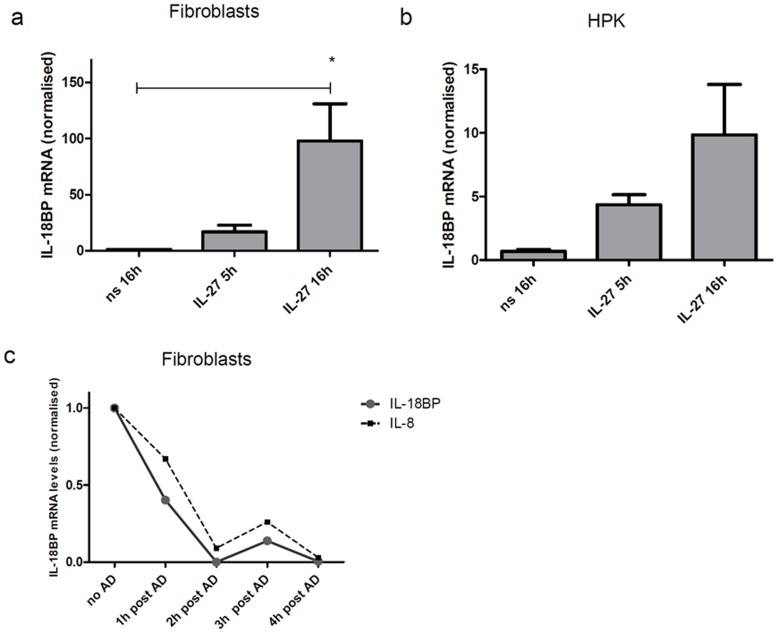
IL-18BP mRNA induction by IL-27. Fibroblasts (a,c) and HaCat cells (b) were stimulated with IL-27 (50 ng/ml) for 5 h or overnight (16 h). qRT-PCR was performed and results were normalised to the expression of the housekeeping gene U6. The result obtained for non stimulated cells (5 h; not depicted) was used as “calibrator” (defined as 1). Stability of the IL-18BP and IL-8 mRNA stability was analysed in IL-27 (50 ng/ml) stimulated cells using actinomycin D (AD). Results were normalised to the expression of the housekeeping gene U6snRNA and the value obtained for cells not treated with AD ( =  no AD) was used as “calibrator” and defined as 1. (a) n = 3, (b) n = 2, (c) one out of 2 independent experiments is depicted. ns = non stimulated.

**Figure 5:**
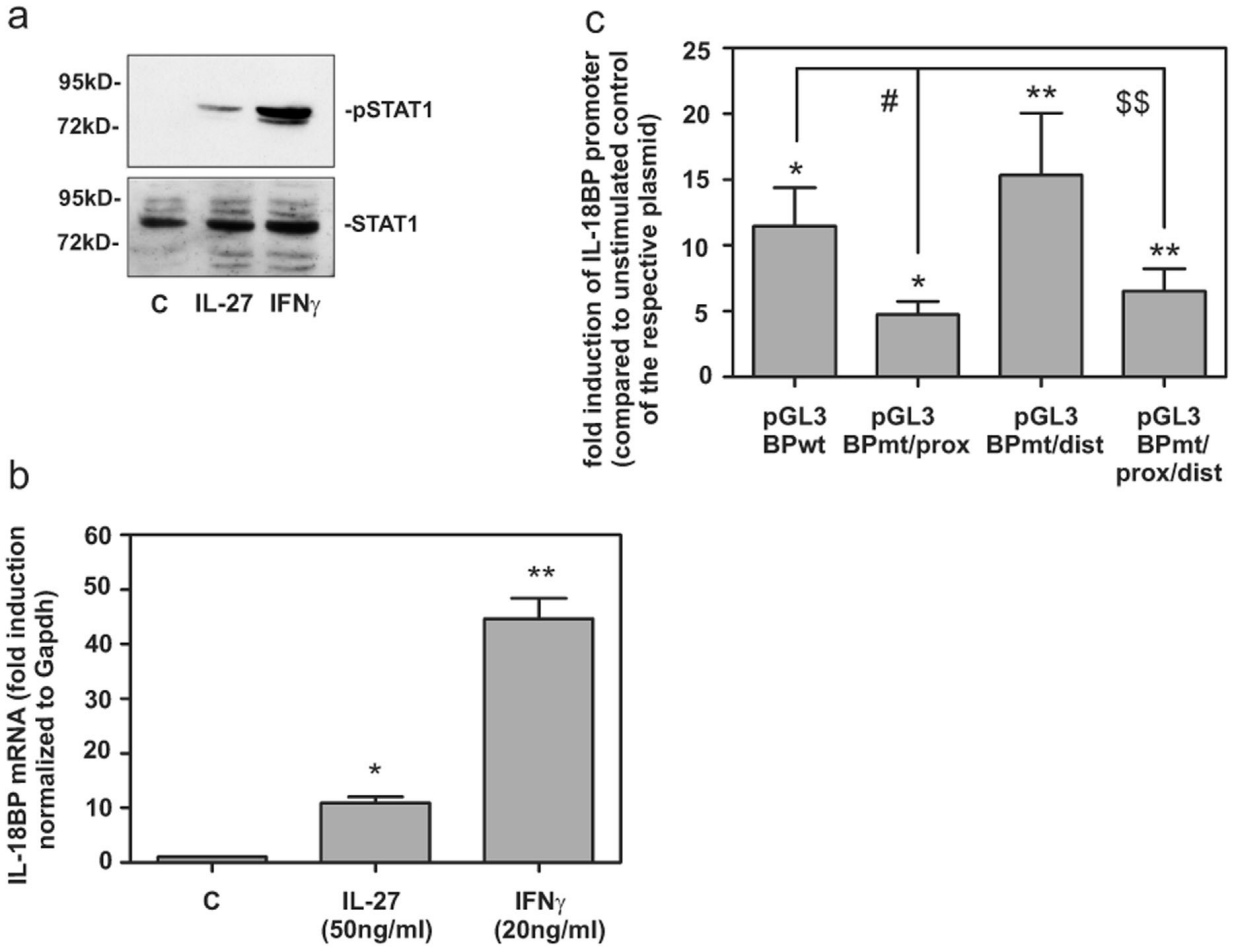
IL-27 induced IL-18BP activation pathway in HaCat cells. (a) HaCat cells were stimulated for 30 min with IL-27 (100 ng/ml), IFNγ (20 ng/ml) or used as non stimulated control, lysed and the obtained nuclear extract analysed by western blot using antibodies specific for total STAT1 and pSTAT1-Y701. One representative of three independently performed experiments is shown. (b) HaCat cells were stimulated for 24 h with with IL-27 (50 ng/ml), IFNγ (20 ng/ml) or used as unstimulated control and mRNA expression of IL-18BP was determined by qRT-PCR. IL-18BP mRNA was normalized to that of GAPDH and is shown as mean fold induction compared to unstimulated control ± S.D. (n = 6). (c) HaCat cells were transfected with the indicated IL-18BP promoter constructs. After 24 h, cells were kept as non-stimulated control or stimulated with IL-27 (100 ng/ml). After another 24 h, cells were harvested and luciferase assays were performed. Data are expressed as mean fold-luciferase induction ± SD (compared to the non-stimulated control transfected with the same plasmid) obtained from 4 independent experiments. *p<0.05 and **p<0.01 compared to non stimulated control of the respective plasmid; ^#^p<0.05 compared pGL3-BPwt under the influence of IL-27, $$p<0.01 compared to pGL3-BPmt/dist under the influence of IL-27.

All cell types showed a robust upregulation of mRNA expression after IL-27 stimulation. The maximum induction after stimulation with either IL-27 or IFNγ occurred with some delay. Keratinocytes and fibroblasts showed higher induction levels after overnight stimulation as compared to 5 h stimulation (Figures 4 A,B and 5B). The inducibility of fibroblasts (fold induction up to 100 fold) was much higher in fibroblasts than in keratinocytes (10 fold in both HPK and HaCat). mRNA stability assays performed with fibroblasts using actinomycin D showed that IL-18BP mRNA was as “unstable” as IL-8 mRNA determined in the same samples (Figure 4C).

In order to further decipher the signalling pathways leading to increased IL-18BP production we analysed HaCat cells which showed a robust induction of IL-18BP similar to HPK. These cells respond to IL-27 with an activation of STAT1 (Figure 5A). STAT1 activationby IL-27 or IFNγ was associated with significant IL-18BP mRNA induction (Figure 5B). Luciferase reporter assays were performed in order to analyse IL-18BP promoter activation under the influence of IL-27. Figure 5C demonstrates induction of the IL-18BP wild type promoter (pGL3-BPwt) in HaCat cells in response to IL-27. Promoter activation was significantly reduced in the context of a mutated proximal GAS site (pGL3-BPmt/prox). By contrast, a dysfunctional distal GAS site (pGL3-BPmt/dist) left the induction unaffected whereas the double mutation (pGL3-BPmt/prox/dist) resulted in similar suppression of the reporter gene activity as the single proximal mutation. These results show that the proximal GAS site at the IL-18BP promoter is crucial for gene activation in response to IL-27.

## Discussion

In most chronic inflammatory skin disease tissue resident cells over-express IL-1 family members, T cell attracting chemokines, proteases (e.g. MMPs) and/or TNFα. One factor for diseases such as cutaneous lupus erythematosus, psoriasis and eczema to become chronic is the disturbed balance in producing pro- and anti-inflammatory molecules by infiltrating leukocytes and tissue cells. An inflammatory response to a given stimulus is normally counter-regulated, once the causing agent is removed. However, in a number of diseases at epithelial surfaces, the pro-inflammatory response is ongoing. IL-18BP may to be of high importance in balancing skin inflammatory responses as supported by the fact that a number skin-tropic viruses induce or express this IL-18 neutralising molecule.

IL-18 seems to play an important pathogenic role with regard to maintaining inflammatory responses. It induces TNFα and favours the release of IFNγ by infiltrating lymphocytes. IL-18 is highly expressed in chronic phases of skin diseases such as eczema, lupus erythematosus and psoriasis but also in e.g. lupus nephritis, chronic joint diseases and graft-versus-host disease [17], [24], [25], [26], [51], [52], [53], [54], [55], [56], [57]. It has been suggested that in allergic contact eczema IL-18 may act upstream of IL-1ß and TNFα in the induction of Langerhans cell migration [58] and its potential role in allergic contact dermatitis is highlighted by the fact that measurement of IL-18 has been suggested as a tool for the identification of substances with high sensitising potential [51]. We lack precise information on the (dys)balanced expression of IL-18 and its natural antagonist IL-18BP in pathological conditions and the need to determine “free” IL-18 activity has been pointed out by Favilli et al. [59]. An elevation of IL-18 and IL-18BP has been described in chronic liver disease by Ludwiczek et al. [60], and the levels reflect the severity of disease. This study suggests that in the patients suffering from advanced cirrhotic disease stages the levels of IL-18BP may not be sufficient to counteract the pro-inflammatory actions of IL-18. Patients with heart failure have also been reported to show increased IL-18 but decreased IL-18BP levels [61].

Basal levels of IL-18BP can be found in the “circulation”. Keratinocytes seem to contribute to a basal level in the skin organ. These cells along with resident and infiltrating APCs do express IL-18. It is important to note, that the here presented data point to dermal fibroblasts as a significant source of inducible IL-18BP production. These cells do not express IL-18. This further highlights the complex interaction between different tissue cell types in maintaining a fine-tuned mediator network balance and highlight fibroblasts as important “regulators”.

Our data support the view that the proximal GAS site in the IL-18BP promoter [47], [48] is crucial for IL-18BP induction. Besides IFNγ, IL-27 is a novel player determining cytokine-induced IL-18BP expression. It has been shown to promote a pro-inflammatory response by priming keratinocytes, macrophages or inflammatory dendritic epidermal cells (IDEC) for TNFα, CXCL10 and IL-23 production respectively, and therefore may contribute to the elicitation of inflammatory skin diseases [6], [7], [8], [13]. On the other hand, it has been shown that IL-27 also suppresses the development of Th1, Th2 and Th17 subsets in later phases of infection. Yoshimura et al. [10] found that IL-27 suppresses the production of e.g. IL-2, IL-4, IFNγ and IL-17 by fully activated CD4+ T cells. These and other findings suggest that in early phases of immune responses, IL-27 may act as an amplifier to achieve a robust response to pathogen associated stimuli, whereas in later phases of immune response, the role of IL-27 seems to be regulatory.

To comprehend the complex actions of IL-27, the understanding of its signalling pathways is crucial. The IL-27 receptor composed of the WSX-1 and the common gp130 chain is widely expressed. Downstream of the IL27R, STAT1 and STAT3 are activated and may coordinate the pleiotropic effects of IL-27. However, it has been shown that STAT3 does not affect IL-18BP expression in colon carcinoma cells [62]. In those cells IL-18BP induction depends in large part on STAT1 binding to the proximal GAS element [48].

In a recent study Murray et al. [49] show that in cardiac cardiomyocytes ß2-adrenergic receptor triggering activates a signalling cascade which ultimately leads to a CREB and c/EBPß dependent increased IL-18BP promoter activity. These findings support data by Hurgin et al. [47] who described c/EBPß as important transcription factor binding to the aforementioned proximal GAS site in the IL-18BP promoter of HepG2 cells. We have stimulated keratinocytes with salbutamol (a beta2 adrenergic agonist) and failed to see any increase in IL-18BP production (data not shown). Taken together these findings indicate that cell type specific differences may exist between primary human tissue cells, murine cells and transfected HepG2 cells with regard to IL-18BP promoter activation. It would be interesting to further investigate potential differences in different cell types and to further elucidate the significance of c/EBPß dependent IL-18BP regulation in human tissues and diseases such as hypertrophic cardiomyopathies.

Our data confirm and expand the current knowledge on IL-18BP regulation in the skin. IL-18BP is highly regulated at transciptional level. We also demonstrate herein the crucial role of the proximal GAS element for IL-18BP promoter activation in response to IL-27. As upon stimulation IL-18BP is produced with some delay (max. production around 48 h after stimulation) but in a prolonged manner (up to 120 h) we were surprised to see that the stability of the mRNA in fibroblasts was not greater than that of IL-8 (known to be “not” stable). However, the protein seem to be rather stable once produced and Hurgin et al. [47] have already pointed to the fact that it accumulates in the supernatants of cultured cells. It has been described that IFNα induces IL-18BP in chronic hepatitis C patients [50]. IL-18BP upregulation by IL-27 could therefore be regulated by endogenous IFNs. In our experimental setup we failed to detect increased IL-27 induced mRNA levels of IFNλ by keratinocytes or fibroblasts and could not detect elevated levels of IFNα in the supernatant of stimulated cells. IFNγ, which is an extremly potent inducer of IL-18BP, is not expressed by human keratinocytes or fibroblasts. We can, however, not fully exclude that type I IFN, namely IFNß, or type III IFN may contribute to the IL-27 effect on IL-18BP upregulation. We seek to investigate how IL-27 interacts with the IFN system in inflammatory skin diseases such as psoriasis and lupus erythematosus in future studies.

It seems of great interest to therapeutically manipulate the IL-18/IL-18BP system. A phase I study [63], has shown that in healthy volunteers, rheumatoid arthritis and psoriasis patients subcutaneous injections of IL-18BP are well tolerated and within 1–2 weeks show steady levels of the protein in serum. This suggests that there is therapeutic viability for this protein. However, efficiacy of the drug in chronic inflammation needs to be further established. In viral infections it seems favourable to reduce IL-18BP expression and thereby to enhance antiviral IL-18 activity. On the contrary, in chronic inflammatory diseases associated with tissue remodelling local counterregulation of IL-18 bioactivity may be highly beneficial. However, increasing IL-18BP by pharmacological means might not be advised under all pathophysiological conditions. In fact, recent data indicate that high levels of IL-18BP, by scavenging immunosuppressive IL-37 [64], [65], may even have a pathogenic pro-inflammatory side. Thus, it appears that tissue IL-18BP needs to be tightly balanced in order to achieve the desired anti-inflammatory effect. IL-27 is an interesting molecule with “regulatory” potential. However, we need to better understand the fine tuned regulation of different effector molecules and the crosstalk between different tissue cells before proposing this molecule as valuable for therapeutic intervention in humans.

## Materials and Methods

### Cytokines and Reagents

All cytokines were used as purified recombinant human preparations. IL-27, IL-12, IL-23 and IFNγ/ß were purchased from eBioscience (Hatfield, UK) or RnD Systems (Abingdon, UK).

### Cell Isolation and Culture

Cultures of human primary keratinocytes (HPK) and fibroblasts were prepared from foreskin as described previously [23]. All patients gave written conformed consent to participate in the study. The procedure to use foreskin from anonymised patients was approved by the Ethical Committee of Hannover Medical School, Hannover, Germany. HPK were cultured in Keratinocyte Growth Medium Kit II (PromoCell, Heidelberg, Germany). HaCat cells as well as fibroblasts were grown in DMEM with 4.5 g/L of glucose and L-Glutamine (Lonza, Slough, UK) supplemented with fetal calf serum (10%) (PromoCell), 0.05 mg/ml streptomycin and 50 U/ml penicillin. Culture medium was changed every second to third day. When the fibroblasts reached 90% confluency (HPK: 60–70% and HaCaT: 70–80% confluency) they were passaged and 20 000 cells were plated into each well of a 24 well plate for stimulation. They were left at least for 24 hours at 37°C after plating before medium was changed and stimulation was carried out. Stimulation of keratinocytes was performed in the absence of hydrocortisone and EGF. For experiment depicted in Figure 5 HaCaT keratinocytes were maintained in DMEM (Invitrogen, Karlsruhe, Germany) supplemented with 100 units/ml penicillin, 100 µg/ml streptomycin, and 10% heat-inactivated FCS (GIBCO-BRL, Eggenstein, Germany). For experiments, HaCat keratinocytes were seeded on 6-well polystyrene plates (Greiner, Frickenhausen, Germany) in the aforementioned culture medium.

### Quantitative Real Time PCR

qRT-PCR for fibroblasts was performed on a RotorGen (Qiagen, Hilden, Germany) using a ΔΔCT-analysis based on the generation of standard curves for both the housekeeping gene (U6snRNA) and the target gene (IL-18BP, QuantiTect Primer Assay, Qiagen). For RNA isolation Quick-RNA MiniPrep (Zymo Research, Cambridge Bioscience, Cambridge, UK) was used. First strand cDNA synthesis kit (Fermentas/Thermo Fisher Scientific, Loughborough, UK) was used for reverse transcription. QuantiFast SYBR green PCR (Qiagen) was used to carry out the RT-PCR.

For IL-18BP mRNA expression in HaCat keratinocytes, total RNA was isolated and transcribed using TRI-Reagent (Sigma-Aldrich), random hexameric primers, and Moloney virus reverse transcriptase (Applied Biosystems, Weiterstadt, Germany) according to the manufacturers’ instructions. During realtime PCR, changes in fluorescence were caused by the Taq-polymerase degrading the probe that contains a fluorescent dye (FAM used for IL-18BP, VIC for GAPDH) and a quencher (TAMRA). Primers and probe for IL-18BPa were designed using Primer Express (Applied Biosystems) according to AF110798: forward, 5′-ACCTCCCAGGCCGACTG-3′; reverse, 5′-CCTTGCACAGCTGCGTACC-3′; probe 5′-CACCAGCCGGGAACGTGGGA-3′. Amplification of genomic DNA was avoided by selecting an amplicon that crosses an exon/intron boundary. For GAPDH pre-developed assay reagents were used (4310884E; Applied Biosystems). Assay-mix was used from Thermo Fisher Scientific. qRT-PCR was performed on AbiPrism 7500 Fast Sequence Detector (Applied Biosystems): One initial step at 95°C for 5 min was followed by 40 cycles at 95°C for 2 s and 60°C for 25 s. Detection, calculation of threshold cycles (Ct values), and data analysis were performed by Sequence Detector software. mRNA was quantified by use of cloned cDNA standards for IL-18BP and GAPDH. Data for IL-18BP were normalized to those of GAPDH.

### Determination of RNA Stability

Fibroblasts were stimulated for 4 h. Actinomycin D (Sigma) was added 30 minutes before stimulation. mRNA expression was monitored for up to 4 h by qRT-PCR. Samples were normalised to U6snRNA which remained stable over the time course measured. QuantiTect Primer Assay for IL-8 was purchased from Qiagen.

### Elisa

Cell-free supernatant was collected, stored at −20 (short term) or −80°C and analysed for the content of IL-18BP using a DuoSet human IL-18BP ELISA kit (RnD Systems, Abingdon, UK) following the manufacturer’s instructions.

### Luciferase Reporter Assay

An IL-18BP promoter fragment was cloned into pGL3-Basic (Promega, Mannheim, Germany) and entitled pGL3-BPwt as previously described [48]. Site directed mutagenesis was performed by using the QuikChange site-directed mutagenesis kit (Stratagene, Amsterdam, Netherlands) in order to generate promoter fragments that show a dysfunctional proximal γ-activated sequence (GAS) (pGL3-BPmt/prox, located at −25 bp to −33 bp), a dysfunctional putative distal GAS site (pGL3-BPmt/dist, located at −625 bp to −633), and a double-mutation of both GAS sites (pGL3-BPmt/dist/prox) as previously described. For each transfection experiment 4 µg of the indicated plasmids were transfected using Nucleofector Technology according to the manufacturer’s instructions (Amaxa, Cologne, Germany). For control of transfection efficiency 0.2 µg pRL-TK (Promega) coding for *Renilla* luciferase were cotransfected. After rest of 24 h, cells were either kept as unstimulated control or stimulated with IL-27 (100 ng/ml). After a 24 h stimulation period, cells were harvested and fold-induction of luciferase activity by IL-27 with control conditions was determined by using the dual reporter gene system (Promega) and an automated chemiluminescence detector (Berthold, Bad Wildbad, Germany) (unstimulated cells transfected with the same respective promoter fragment) set to 1.

### Western Blot

To detect total STAT1 and activated phosphorylated pSTAT1, nuclear extracts of HaCat cells were isolated as previously described [66]. For detection of total nuclear STAT1, blots were stripped and reprobed. Antibodies: Total STAT1, rabbit polyclonal antibody (Santa Cruz Biotechnology, Heidelberg, Germany); pSTAT1-Y701, rabbit polyclonal antibody (Cell Signaling, Frankfurt, Germany).

### Statistical Analysis

Raw data were analysed by non-paired student’s t-test (GraphPad Prism 5.03, GraphPad Software, San Diego, CA).
